# Efficacy of Allogeneic Hematopoietic Stem Cell Transplantation in Intermediate-Risk Acute Myeloid Leukemia Adult Patients in First Complete Remission: A Meta-Analysis of Prospective Studies

**DOI:** 10.1371/journal.pone.0132620

**Published:** 2015-07-21

**Authors:** Dandan Li, Li Wang, Honghu Zhu, Liping Dou, Daihong Liu, Lin Fu, Cong Ma, Xuebin Ma, Yushi Yao, Lei Zhou, Qian Wang, Lijun Wang, Yu Zhao, Yu Jing, Lili Wang, Yonghui Li, Li Yu

**Affiliations:** 1 Department of Hematology, Chinese PLA General Hospital, Beijing, China; 2 Medical College of Chinese PLA, Beijing, China; 3 Department of Hematology, Peking University People’s Hospital, Peking University Institute of Hematology, Beijing, China; 4 Department of Hematology, Peking University Third Hospital, Beijing, China; 5 Department of clinical laboratory, PLA Navy General Hospital, Beijing, China; 6 Tumor diagnosis and treatment center, PLA Navy General Hospital, Beijing, China; 7 Department of Hematology, No. 202 Hospital of PLA, Shenyang, China; Hospital Infantil Universitario Niño Jesús, SPAIN

## Abstract

Hematopoietic stem cell transplantation (HSCT) and consolidation chemotherapy have been used to treat intermediate-risk acute myeloid leukemia (AML) patients in first complete remission (CR1). However, it is still unclear which treatments are most effective for these patients. The aim of our study was to analyze the relapse-free survival (RFS) and overall survival (OS) benefit of allogeneic HSCT (alloHSCT) for intermediate-risk AML patients in CR1. A meta-analysis of prospective trials comparing alloHSCT to non-alloHSCT (autologous HSCT [autoHSCT] and/or chemotherapy) was undertaken. We systematically searched PubMed, Embase, and the Cochrane Library though October 2014, using keywords and relative MeSH or Emtree terms, ‘allogeneic’; ‘acut*’ and ‘leukem*/aml/leukaem*/leucem*/leucaem*’; and ‘nonlympho*’ or ‘myelo*’. A total of 7053 articles were accessed. The primary outcomes were RFS and OS, while the secondary outcomes were treatment-related mortality (TRM) and relapse rate (RR). Hazard ratios (HR) and 95% confidence intervals (CI) were calculated for each outcome. The primary outcomes were RFS and OS, while the secondary outcomes were TRM and RR. We included 9 prospective controlled studies including 1950 adult patients. Patients with intermediate-risk AML in CR1 who received either alloHSCT or non-alloHSCT were considered eligible. AlloHSCT was found to be associated with significantly better RFS, OS, and RR than non-alloHSCT (HR, 0.684 [95% CI: 0.48, 0.95]; HR, 0.76 [95% CI: 0.61, 0.95]; and HR, 0.58 [95% CI: 0.45, 0.75], respectively). TRM was significantly higher following alloHSCT than non-alloHSCT (HR, 3.09 [95% CI: 1.38, 6.92]). However, subgroup analysis showed no OS benefit for alloHSCT over autoHSCT (HR, 0.99 [95% CI: 0.70, 1.39]). In conclusion, alloHSCT is associated with more favorable RFS, OS, and RR benefits (but not TRM outcomes) than non-alloHSCT generally, but does not have an OS advantage over autoHSCT specifically, in patients with intermediate-risk AML in CR1.

## Introduction

Acute myeloid leukemia (AML) is a heterogeneous disease. An important prognostic factor for AML patients is the presence of cytogenetic abnormalities at diagnosis. The categories of AML (good-, intermediate-, and poor-risk), based on cytogenetic features have each been assigned a risk-adapted treatment regimen after post-remission therapy [1]. According to the AML guidelines of the National Comprehensive Cancer Network (NCCN; AML, Version 1.2014; www.nccn.org) [2], high-dose cytarabine (HiAra-C)-based chemotherapy is most beneficial for patients with core-binding factor AML[3,4]. Allogeneic hematopoietic stem cell transplantation (alloHSCT) has been established as the preferred post-remission therapy for AML patients with defined adverse risk cytogenetic features [5–7]. However, the best post-remission treatment (whether alloHSCT, or non-alloHSCT [autologous stem cell transplantation (autoHSCT), chemotherapy]) for intermediate-risk AML patients remains to be determined [8–10].

Over the past four decades, there has been evidence demonstrating the efficacy of HSCT in patients with intermediate-risk AML. According to donor versus no-donor studies, alloHSCT is the best treatment option for younger patients with intermediate-risk AML in first complete remission (CR1) [9,11], as it confers a significant relapse-free survival (RFS) and overall survival (OS) benefit in these patients [12]. In contrast, another study showed that there was no RFS or OS benefit [5]. Moreover, numerous prospective trials have demonstrated that alloHSCT increases treatment-related mortality (TRM) [5,11,12], and can lead to graft-versus-host disease (GVHD), which has substantial adverse effects on the quality of life.

With advances in determining the cytogenetic and molecular lesions underlying the pathogenesis of AML, risk-stratified treatment has become possible. There is evidence that cytogenetic analysis can identify biologically distinct subsets of AML, allowing tailored therapeutic approaches [13,14]. Moreover, higher resolution and key loci tested for HLA matching [15], the increase in unrelated-donor pool sizes, and the use of haplo-identical HSCT technology [16,17] have improved donor HLA matching and selection. There have also been improvements in conditioning regimens, supportive relative therapy (including carbapenem and antifungal agents to treat bacterial and fungal infections), and new immune suppressant drugs such as tacrolimus and mycophenolate mofetil for GVHD prophylaxis [18,19]. Technological improvements have been aided by an increase in the number of alloHSCT clinical trials that have been carried out to determine the optimal post-remission treatment for intermediate-risk AML. Hence, we asked whether using alloHSCT to treat intermediate-risk AML patients in CR1 was comparable to using autoHSCT. If autoHSCT has similar RFS and OS benefits to alloHSCT in these patients, it would be highly valuable information because the autograft source is easier to obtain and is associated with fewer less post-transplant complications, especially GVHD.

Koreth et al. [9] carried out a meta-analysis to analyze alloHSCT for AML patients, and included good-, intermediate-, and poor-risk subgroup analysis. As they only analyzed RFS and OS, there were no overall robust data on TRM and relapse rate (RR). For intermediate-risk AML patients in CR1, the doctor should balance disease-related and transplant-related risks before their decision make. Unfortunately, there are currently no uniform guidelines. In our study, we pooled the primary outcomes (OS and RFS) and the secondary outcomes (TRM and RR) of available prospective clinical trial data.

## Methods

We searched PubMed, Embase and the Cochrane Library Registry of Controlled Trials (updated October 2014), using the following terms and related MeSH terms: ‘allogeneic’; ‘acut*’ and ‘leukem*/ aml/ leukaem*/ leucem*/ leucaem*’; and ‘nonlympho*’ or ‘myelo*’, which is the search strategy used by Koreth et al. [9]. We limited our search to adults, humans, and English and Chinese language articles. The titles and abstracts were screened, and non-relevant articles were excluded. Cross-references from selected articles, recent reviews, and meta-analysis were also accessed to identify other potentially eligible studies [9,20,21]. Full text articles were assessed to extract the data for this meta-analysis.

Potential studies for inclusion were prospective trials of adults (wholly or largely) with intermediate-risk AML in CR1 that were assigned to receive alloHSCT or non-alloHSCT. The intermediate-risk classification was defined by cytogenetics and molecular abnormalities. The outcomes were OS, RFS, RR, and TRM. If more than one publication reported a trial, the most up-to-date data were analyzed. Unadjusted hazard ratios (HR) were recorded in our analysis, as adjusted HR values may have been modified according to different variables in different studies. The baseline characteristics were assessed to equalize related covariates between the alloHSCT and non-alloHSCT groups. Furthermore, we utilized the Newcastle-Ottawa Scale to determine the quality of the included articles [22].

Two reviewers independently extracted the data. Data were recorded included the following: first author, publication year, total patient numbers, number of patients assigned to each treatment category, median follow-up duration (months), number of events (death and relapse) in each arm, assessment criteria for intermediate-risk AML, induction treatment, conditioning regimen, study endpoints for OS, and/or RFS benefit and so on. We recorded OS and RFS (also reported as disease free survival, failure-free survival, or leukemia-free survival) according to the individual studies. Data on RR and TRM (also reported as non-relapse mortality) were also collected. If important information was not provided in the paper, we attempted to contact the corresponding author to obtain it.

We used Stata (version 12.0) software (StataCorp, College Station, TX) to analyze the data. Publication bias was estimated using a funnel plot and *P* values from the Egger’s test. The Q statistic and I^2^ were used to assess heterogeneity. Some of the HRs for RFS and OS were calculated using the spreadsheet [23]. A forest plot with pooled HRs and 95% confidence intervals (CI) for the RFS, OS, TRM, and RR benefit of alloHSCT versus non-alloHSCT was used in random effects analysis, regardless of the heterogeneity between groups. Further subgroup analysis of OS was conducted. *P* < 0.05 was considered statistically significant. To evaluate the impact of missing RFS or OS data, we conducted sensitivity analyses. We also analyzed the impact of trials that stratified treatment options according to subgroup, such as alloHSCT versus autoHSCT and alloHSCT versus chemotherapy.

## Results

### Study Selection and Characteristics

**Fig 1:**
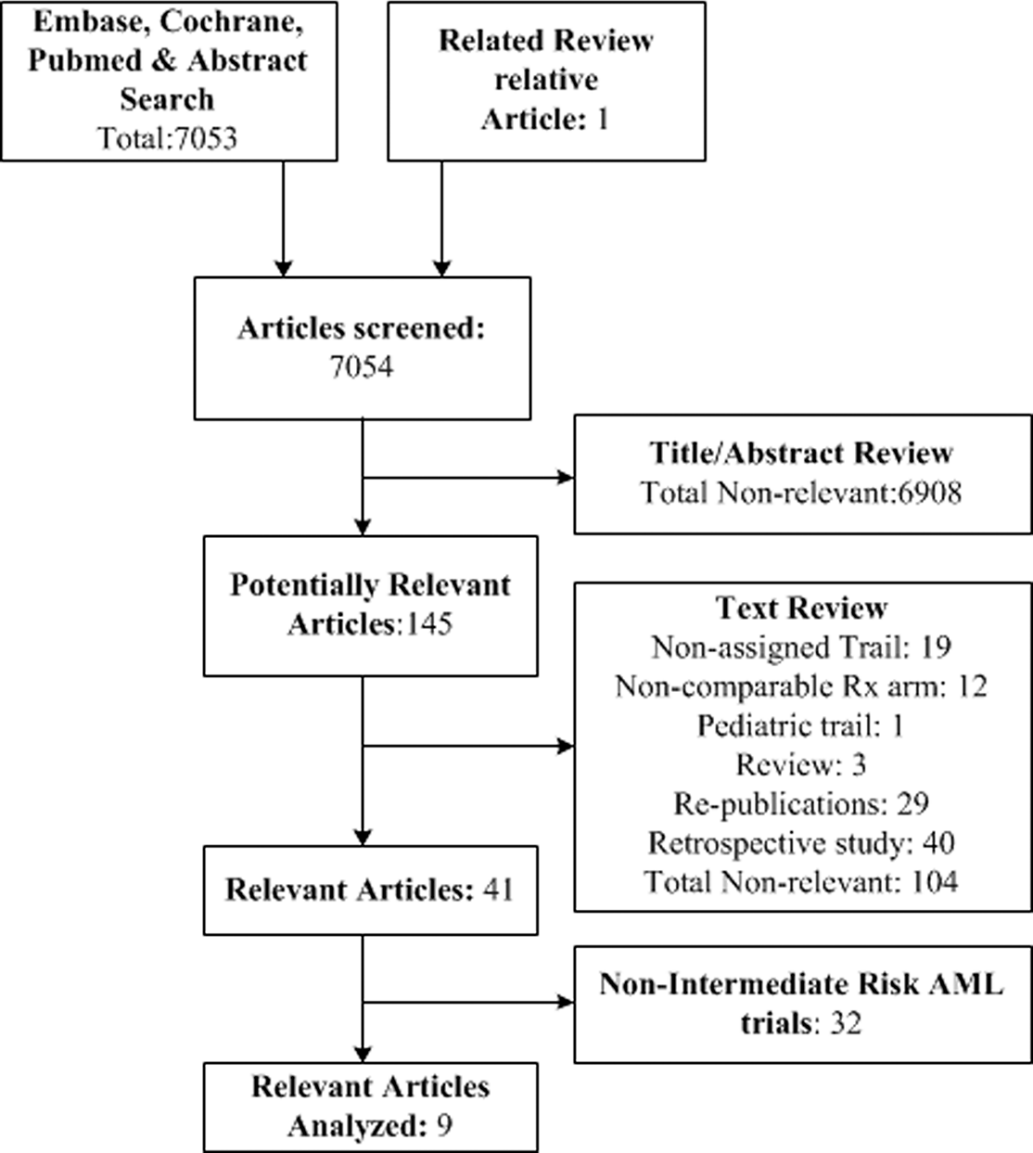
The flowchart of search strategy.

Our initial online search yielded 7053 articles (Fig 1). A total of 6908 non-relevant articles were excluded after screening the titles and abstracts. Two reviewers carefully read 145 full text articles in a structured format. A total of 41 articles relevant to autoHSCT versus non-alloHSCT treatment for AML in CR1, including 9 articles that referred to intermediate-risk AML in CR1, were selected. We recorded 41 articles relevant to alloHSCT versus non-alloHSCT treatment for AML patients in CR1 that provided prospective data on RFS and/or OS [7,24–54], as detailed in S1 Table. Of these, 9 articles were related to intermediate-risk AML [5,6,11,12,55–59]; therefore, we extracted these data in detail (Tables 1, 2, 3 and 4). When estimating and extracting the data, there were no significant discrepancies between the analyses of the two reviewers. It is noted that the “intermediate-risk” acute myeloid leukemia is not a general consensus group, it is a dynamic changing concept and the included articles involved different “intermediate-risk” definition. However, the majority of “intermediate-risk” AML is identical and the risk stratification based on cytogenetics and molecular abnormalities is same, that is “intermediate-risk” includes normal cytogenetics, +8, and all other abnormal cytogenetics. Thus, we think it is feasible to pool these articles. To better show the concept of “intermediate-risk” AML evolves, we summarized the change of intermediate-risk (Table 3). Based on the cytogenetics and molecular abnormalities of intermediate-risk changes recently, we classified the 9 included articles into two subgroups: earlier criteria group and updated criteria group, and conducted a subgroup meta-analysis based on this clinical heterogeneity.

**Table 1:** Summary of trials characteristic.

Author Publication y	Trial Name	N	Enrollment ys	AlloHSCT Arm Median age y (range)	Non-alloHSCT arm Median age y (range)	Int-risk group Median age y (range)	Median follow-up Mon (range)
Harousseau 1997^a^[55]	GOELAM	94	1987–1994	NA	NA	NA	62 (23–103)
Slovak 2000[6]	E3489/S9034	128	1990–1995	34 (18–54)	39 (16–55)	40 (16–55)	57.6 (8–90)
Suciu 2003[5]	EORTC/GIMEMA-AML10	165	1993–1999	35 (15–45)	33 (15–45)	NA	48 (NA)
Tsimberidou2003[56]	AML8	49	1996–2000	mean: 28 ~^b^	Mean: Auto 44 (NA)^b^Mean: Chemo 46 (NA)^b^	NA	43 (18–64)
Brunett 2006[12,64]	MRC AML10	713	1988–1995	NA (0–45+)	NA (0–45+)	NA	142 (26–193)
Cornelissen 2007[11]	HOVON/SAKK AML4/29/42	511	1987–2003	39 (15–55)	39 (16–55)	NA	63 (NA)
Pfirrmann 2012[57]	AML96-1	190	1996–2003	41 (15–60)	Auto 47 (17–60)Chemo 50 (18–60)	48 (42–56)	98.4 (3.6–162)
Zhu 2013[58]	AML05	32	2005–2011	38 (15–53)^b^	28 (14–59)^b^	36.5 (14–59)	36 (6–83)
Stelljes 2014[59]	AMLCG 99	68	1999–2011	45 (16–59)	46 (17–59)	NA	94.8 (NA-144)

y indicates year; Int-risk, intermediate-risk; NA, not applicable; Auto, autogenetic group; and Chemo, chemotherapy.

^a^This study only reported 4y-RFS and 4y-OS.

^b^data from int-risk AML CR1 group.

**Table 2:** Therapies utilized of trials.

Author Publication y	Induction Therapy (optional)	Consolidation Chemotherapy	Conditioning Regimen
Harousseau 1997[55]	Ara-C+IDR/RBZ	Amsa+Ara-C	Bu+Cy; TBI
Slovak 2000[6]	IDA+Ara-C×1–2	HiAra-C	Bu+Cy
Suciu 2003[5]	DNR/IDA/Mito+Ara-C+VP×1–2	DNR/IDA/Mito+Ara-C+VP×1–2	Cy+TBI (12Gy); Bu+Cy
Tsimberidou 2003[56]	Ara-C+IDA×2	HiAra-C	Bu+Cy
Brunett 2006[12,64]	DNR+Ara-C+Tg/VP×2	CTX	Cy+TBI (7.5-14Gy); Bu+Cy
Cornelissen 2007[11]	DNR/IDA+Ara-C-> Amsa+midAra-C	CTX+Mito+etoposide (only 65%)	Bu+Cy
Pfirrmann 2012[57]	MidAra-C; Mito, etoposide and Amsa×2	HiAra-C	TBI (12Gy); Bu+Cy
Zhu 2013[58]	DNR+IDA×1–2	MidAra-C±DNR/Mito	Ara-C+Bu+Cy+Me-CCNU±ATG
Stelljes 2014[59]	Tg+Ara-C+DNR or HiAra-C+Mito×2	Tg+Ara-C+DNR or none	Bu+Cy

y indicates year; NA, not applicable; Cy, cyclophosphamide; TBI, total body irradiation; Bu, busulfan; Ara-C, cytarabine; IDR, idarubicin; RBZ, rubidazone; Amsa, amsacrine; IDA, idarubicin; HiAra-C: high-dose Ara-C; DNR, daunorubicin; Mito, mitoxantrone; VP, etoposide; Tg, thioguanine; CTX, cyclophosphamide; MidAra-C, intermediate-dose AraC; Mel, melphalan; Flud, fludarabine; BUS, busulfan; and ATG, Anti-thymocyte globulin.

**Table 3:** Eligibility, intermediate-risk criteria and other characteristic of trials.

Author Publication y	Multi-center	Eligibility for Study	Standard criteria	Intermediate-risk inclusion	Stem cellsource	Donor category
Harousseau 1997[55]	Yes	de novo AML; 15–50y	NA	All other abns excluding: t(8;21), t(15;17) or inv (16), -5, 5q-, -7, or multiple abns	BM	MSD
Slovak 2000[6,9]	Yes	AML; 16–55y; no prior treatment; no infection/renal/hepatic/cardiac diagnosis	SWOG	+8,-Y, +6, del(12p), or NK	BM	MSD or HLAsingle mismatched family donor
Suciu 2003[5,9]	Yes	AML; 15–46y; no prior Rx/MDS/APL; no renal/hepatic/cardiac/pulmonary/neurologic diagnosis	ISCN	NK,-Y	BM (some TCD)	MSD
Tsimberidou 2003[56]	Yes	de novo AML; ≤60y; no APL or M3v; performance status score≤2; no hepatic/cardiac/infection diagnosis	NA	NK (+8 or <3 abns), excluding those involving chromosomes 5 or 7	BM	MSD
Brunett 2006[9,12,64]	Yes	AML; ≤55y includes pediatric; few "good-risk cytogenetics"	NA	NK, all other abns excluding: t(15;17), t(8;21), inv(16); -7, -5, del 5q, abn(3q) and CK	BM	MSD
Cornelissen 2007[11]	Yes	de-novo AML; 15–50 or 55y; no APL; no severe metabolism/cardiac/pulmonary/neurologic diagnosis	NA	All other abns excluding: t(8;21)(q22;q22), inv(16), t(16;16)(p13; q22), nor CK, -5q, -7q, abn(3q), t(6;9)(q23;q34), abn(11q23), t(9;22)(q34;q11)	BM	MSD
Pfirrmann 2012[57]	Yes	15–60y; de-novo or secondary AML; CR; excluding t(8;21)AML	NA	Except the following karyotypes: CK, -5/del(5q), -7/del(7q), hypodiploid karyotypes (other than-X and-Y), abn3q, abn11q, abn12p, t(6;9), t(9;22), t(9;11), +11, +13, +21, or +22. Including inv(16)/t(16;16)	BM/PB	MSD
Zhu 2013[58]	Yes	14–60y; de-novo AML with t(8;21); received CR with one or two induction cycles; no contraindications	NCCN14	t(8;21)AML with c-KIT mutation	BM+PB	MSD, MUD, HRD
Stelljes 2014[59]	Yes	de-novo AML, ≥16 ys, MDS with more than 10% BM blasts	ELN-2010	Cytogenetic abns not classified as favorable or adverse	BM/PB	MSD, MUD

y indicates year; NA, not applicable; BM, bone marrow; MSD, HLA-matched sibling donor; abns, abnormality; NK, normal karyotype; TCD, T-cell depleted; CK, complex karyotype; PB, peripheral blood; MUD, HLA-matched unrelated donor; and HRD, haploidentical related donor.

**Table 4:** The comparison and outcome of alloHSCT benefit in intermediate-risk AML-CR1?

Author Publication y	AlloHSCT v Non-HSCT Arms	Overall conclusion in AML		Overall conclusion in int-risk AML				Allo v Auto in int-risk AML		Allo v CC in int-risk AML	
		RFS	OS	RFS	OS	TRM	RR	RFS	OS	RFS	OS
Harousseau 1997[55]	Allo v CC	No		No^a^	No^a^						
Slovak 2000[6]	Allo v Auto v CC	No	No		No				No		No
Suciu 2003[5]	Allo v Auto	Yes	No	No	No	Yes	No	No	No		
Tsimberidou 2003[56]	Allo v Auto v CC			No	No			No	No		No
Brunett 2006[12,64]	Allo v Auto v Obs	Yes	No	Yes	Yes	Yes	Yes				
Cornelissen 2007[11]	Allo v Auto v Obs	Yes	No	Yes	No	Yes	Yes				
Pfirrmann 2012[57]	Allo v Auto v CC				Yes				Yes		Yes
Zhu 2013[58]	Allo v Auto/CC			No	No	No	No				
Stelljes 2014[59]	Allo v CC	Yes	Yes	Yes	Yes					Yes	Yes

y indicates year; Int-risk, intermediate-risk; Allo, allogeneic stem cell transplantation; Auto, autologous stem cell transplantation; and CC, consolidation chemotherapy.

^a^The studies data were not analyzed in this meta-analysis, for there were no available data for HR and 95% CI, only reported outcome.

The empty tables show there were not applicable data.

### Qualitative Assessment

The articles included in our review were regarded as high quality, as the main inclusion criteria were that the trial had to be prospective and controlled to avoid confounding errors of bias that occur with retrospective analyses. The clinical trials enrolled patients, ranging from 32 to 713 in number, from 1987 to 2011. The trial that included only 32 patients was not excluded from the study because it was based on the new cytogenetic criteria [58]. The inclusion criteria for patients were as follows: de-novo adult AML, no severe metabolic disease, and no cardiac, pulmonary, or other diseases (Table 2). One of the 9 articles included some pediatric patients [12], another included a minority population with myelodysplasia syndrome [59], and a third included patients with secondary AML [57]. Different studies had varying cytogenetic criteria, such as those of the Southwest Oncology Group (SWOG), International System for Cytogenetic Nomenclature (ISCN), the Medical Research Council (MRC United Kingdom), and the NCCN 2014 (Table 3). We summarized the details according to the main cooperative group cytogenetic risk categories mentioned in our inclusion studies. In the trial conducted by Pfirrmann et al. [57], there were three points of relevance to consider. First, they included some intermediate-risk and inv(16)/t(16;16) patients because they had used their own criteria to categorize the patient group. Second, the intermediate-risk group in this study was based on an estimate. According to the cytogenetic risk profile at diagnosis, there were 469 intermediate-risk, and 91 high-risk AML patients, but in the final analysis, the author included 452 cases with complete data. Therefore we assumed that the majority of patients had intermediate-risk AML. Third, when extracting the data related to intermediate-risk, we chose the favorable score groups (AML96). We did not include AML2003 trials, because the population studied in this trial was not equivalent to that of AML96; it included good-, intermediate-, and poor-risk patients, not just intermediate- and poor-risk patients. We used the Newcastle-Ottawa Scale to comprehensively assess each of the 9 studies included (Tables 5 and 6) [22]. These scale tables, includes the most important factors to be compared, as well as the other factors. However, we did not strictly abide by the important or the other factors that needed to compare; instead, we only described the baseline characteristics (Table 6).

**Table 5:** The selection of Newcastle-Ottawa Scale.

Author Publication y	Representativeness of the exposed cohort (a or b = 1, c or d = 0)	Selection of the not exposed cohort (a = 1)	Ascertainment of exposure (a or b = 1)	Demonstration that outcome of interest was not present at start of study (a = 1, b = 0)
Harousseau 1997[55]	b	a	a	a
Slovak 2000[6]	b	a	a	a
Suciu 2003[5]	b	a	a	a
Tsimberidou 2003[56]	a	a	a	a
Brunett 2006[12,64]	a	a	a	a
Cornelissen 2007[11]	a	a	a	a
Pfirrmann 2012[57]	b	a	a	a
Zhu 2013[58]	b	a	a	a
Stelljes 2014[59]	a	a	a	a

y indicates year.

**Table 6:** The comparison and outcome of Newcastle-Ottawa Scale.

Author Publication y	Comparability of cohorts on the basis of the design or analysis^a^	Assessment of outcome (a or b = 1)	follow-up long enough (a = 1, b = 0)	Adequacy of follow up of cohorts (a = 1, b = 0)
Harousseau 1997[55]	NA	b	a	b
Slovak 2000[6]	NA	b	a	a
Suciu 2003[5]	Age, WBC count at diagnosis, FAB subtype, and the CR rate after the first induction course^c^	b	a	a
Tsimberidou 2003[56]	Age^b^	b	a	a
Brunett 2006[12,64]	Age, Sex, Type of AML, WBC count, FAB type,Risk group, Status after course 1, Intermediate-risk, Adverse-risk, Unknown^c^Favorable-risk^b^	b	a	a
Cornelissen 2007[11]	Age, FAB type, WBC count, Number of cycles to achieve remission, Cytogenetic risk distributions prognostic risk score^b^	b	a	a
Pfirrmann 2012[57]	only described: age, sex, WBC count, disease status, cytogenetic risk profile at diagnosis, combined cytogenetic risk, disease status variable, FLT3-ITD mutant-to-wild-type ratio, NPM1 mutation status, CEBPA mutation status, peroxidase-positive blasts, CD34-positive blasts, Blasts in bone marrow after first cycle of induction	b	a	b
Zhu 2013^d^[58]	Age, WBC count, BM blast^c^	b	a	a
Stelljes 2014[59]	Age, cytogenetic risk classification, sex, FAB type, WBC count, LDH, induction treatment^c^	b	a	a

y indicates year; NA, not applicable; and WBC, white blood cell.

^a^For most of the intermediate-risk are subgroup of the AML patients, there are no direct comparison between intermediate-risk group, so this item we just referred, not literally to the criteria

^b^ *P* < 0.05

^c^ *P* > 0.05

^d^comparison of group among intermediate-risk AML patients, other comparison of AML patients.

Because our aim was to analyze outcomes following alloHSCT and non-alloHSCT, we included all prospective controlled studies, including donor versus no-donor trials and other forms of trials. We could not assess the potential bias produced by patient selection and the exclusion of patients with no HLA-matched siblings.

To ensure relative comparability, the 9 studies included in our meta-analysis had similar induction, consolidation chemotherapy, and conditioning regimens (Table 2). The induction regimens in most cases were daunorubicin and cytarabine (the DA regimen) or different doses of cytarabine, while the consolidation regimen was mainly cytarabine with or without other drugs. The myeloablative regimen included busulfan (Bu) and cyclophosphamide (Cy) or total body irradiation (TBI), followed by graft infusions (bone marrow and/or peripheral blood stem cells). When extracting the data, the essential requirement was that any heterogeneity within the study not be significant. There was one study where the patients who underwent alloHSCT were younger than those who underwent non-alloHSCT (autoHSCT or HiAra-C) [56]. It is important to note that the aim of this meta-analysis was to study intermediate-risk AML. Indeed, some studies did not have clinical characteristics of subgroup comparing alloHSCT with non-alloHSCT arms. Table 4 listed the outcome of alloHSCT benefit or not in intermediate-risk AML-CR1.

### RFS benefit

**Fig 2:**
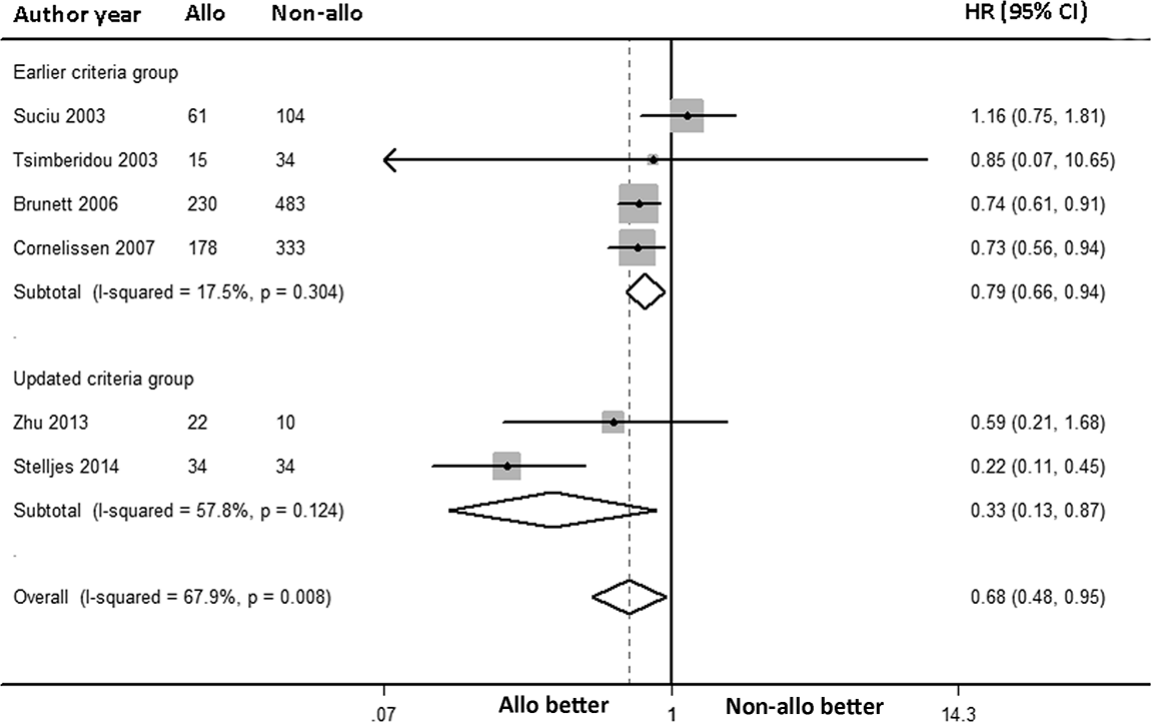
Forest plot of the RFS benefit of alloHSCT in intermediate-risk AML-CR1.

The overall RFS was analyzed via a random-effects forest plot of the HRs from all of the studies. A total of 7 articles reported intermediate-risk AML data for RFS, while only 1 article reported 4-year RFS. The overall HR was 0.68 [95% CI: 0.48, 0.95] (*P* = 0.024). For the 6 articles, adjusted HRs and non-adjusted HRs were pooled, and I^2^ was 67.9% (*P* = 0.008; Fig 2). AlloHSCT-treated intermediate-risk AML patients in CR1 had a significant decrease in the incidence of death or AML relapse. The recent two articles [58,59] included “intermediate-risk” AML patients had a minor difference definition with the others. Then, we conducted a subgroup meta-analysis based on this clinical heterogeneity. The result showed both RFS benefit of alloHSCT in earlier criteria group and updated criteria group (HR: 0.79, 95% CI: 0.66 to 0.94; HR: 0.33, 95% CI: 0.13 to 0.87; respectively, Fig 2).

### OS benefit

**Fig 3:**
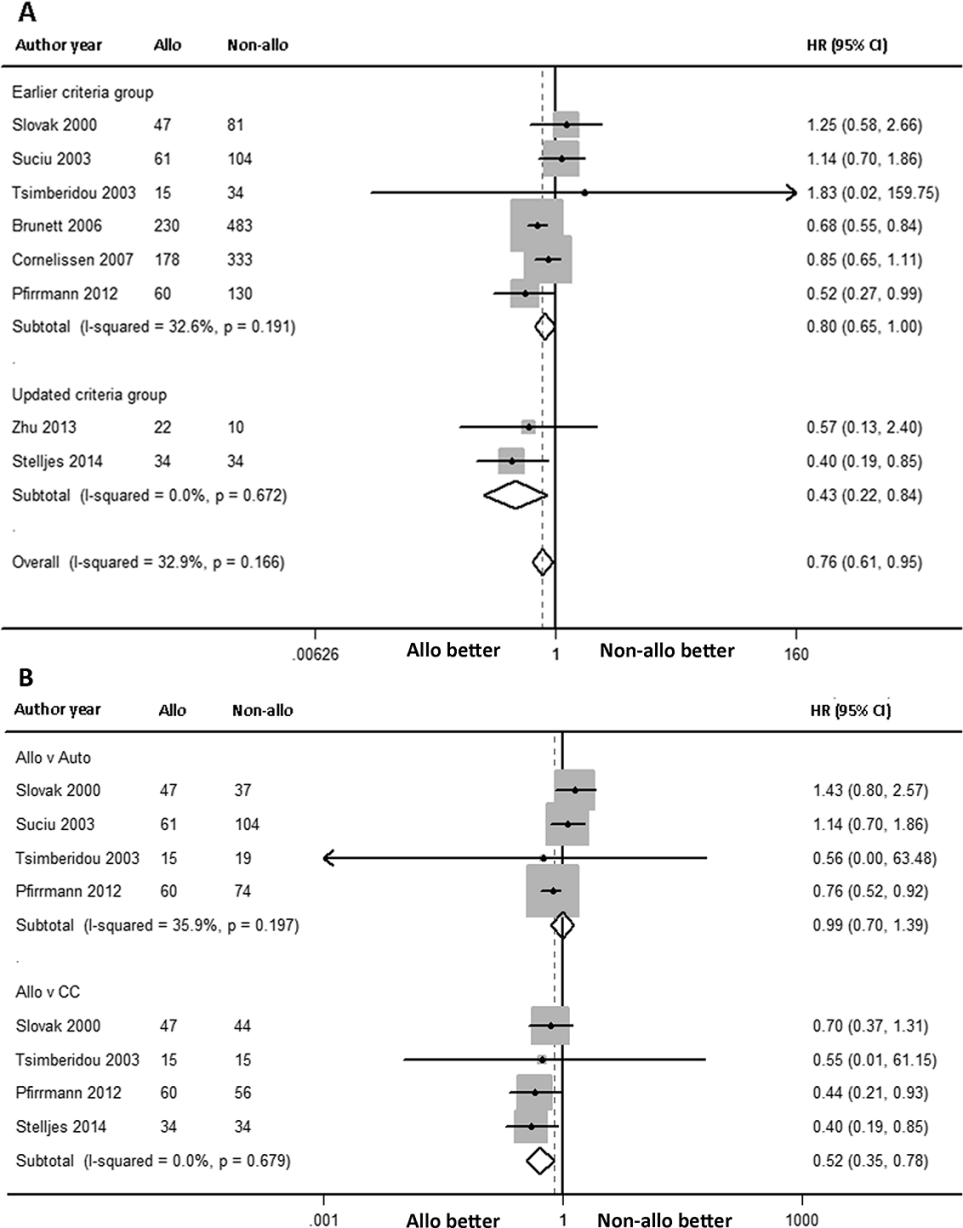
Forest plot of the OS benefit of alloHSCT in intermediate-risk AML-CR1. (A) Forest plot of the overall OS benefit and the subgroup OS benefit (earlier criteria group versus updated criteria group) in intermediate-risk AML-CR1. (B) Forest plot of the subgroup OS benefit (alloHSCT versus autoHSCT, alloHSCT versus chemotherapy) in intermediate-risk AML-CR1. The study of Tsimberidou 2003 has a wide 95% CI, we speculated it may influence by the small size of number.

The OS was analyzed via a random-effects forest plot of the HRs from all of the studies. A total of 9 articles reported intermediate-risk AML data for OS, including some articles that reported the adjusted OS. However, only 1 article reported 4-year OS, so this article was not included in the final analysis. The overall HR was 0.76 [95% CI: 0.61, 0.95] (*P* = 0.016), and the overall I^2^ was 32.9% (*P* = 0.166; Fig 3A). Data were available to compare alloHSCT and autoHSCT subgroups as well as alloHSCT and chemotherapy subgroups. The former included 4 articles with 183 and 234 patients, respectively, while the latter included 4 articles assessing 156 and 149 patients, respectively. Interestingly, we found that the HR of OS for alloHSCT versus autoHSCT was 0.99 [95% CI: 0.70, 1.39] (*P* = 0.944) (I^2^ = 35.9%, *P* = 0.197, Fig 3), while that for alloHSCT versus chemotherapy was 0.52 [95% CI: 0.35, 0.78] (*P* = 0.001) (I^2^ = 0.0%, *P* = 0.679, Fig 3B). This indicated that alloHSCT did not confer an OS benefit over autoHSCT; however, an OS benefit with alloHSCT compared to chemotherapy was noted. Subgroup meta-analysis based on previous mentioned clinical heterogeneity showed the differences between alloHSCT and non-alloHSCT were not significant in earlier criteria group (HR: 0.80, 95% CI: 0.65 to 1.00, Fig 3A), however, alloHSCT has OS benefits compared to non-alloHSCT in updated criteria group (HR: 0.43, 95% CI: 0.22 to 0.84, Fig 3A). It is consisted with overall conclusion.

### TRM benefit

**Fig 4:**
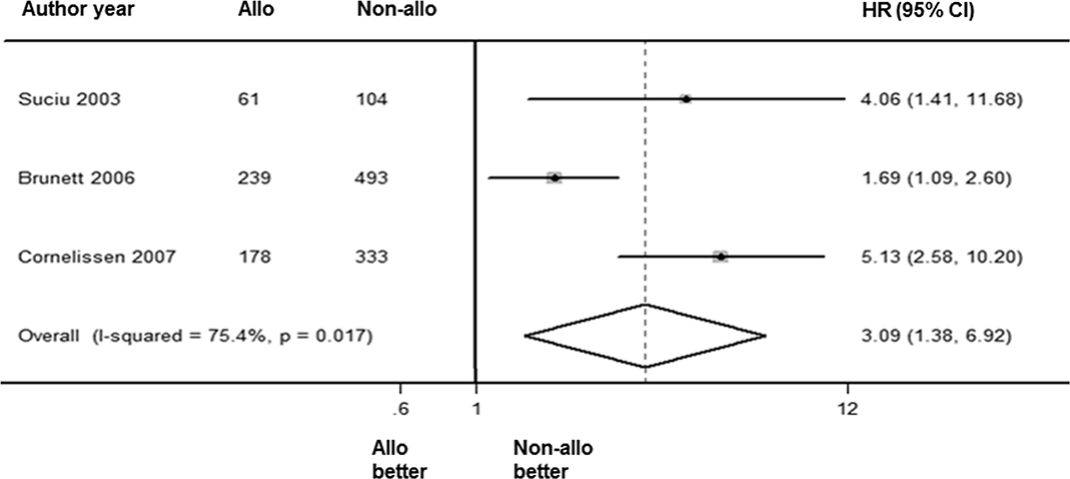
Forest plot of TRM benefit of alloHSCT in intermediate-risk AML-CR1.

The overall TRM was analyzed via a random-effects forest plot of HRs from all of the studies. A total of 4 articles reported intermediate-risk AML data for TRM. The overall HR was 3.09 [95% CI: 1.38, 6.92] (*P* = 0.006). The overall I^2^ was 75.4% (*P* = 0.017; Fig 4). This outcome indicated that the alloHSCT group had higher non-relapse mortality than the non-alloHSCT group.

### RR benefit

**Fig 5:**
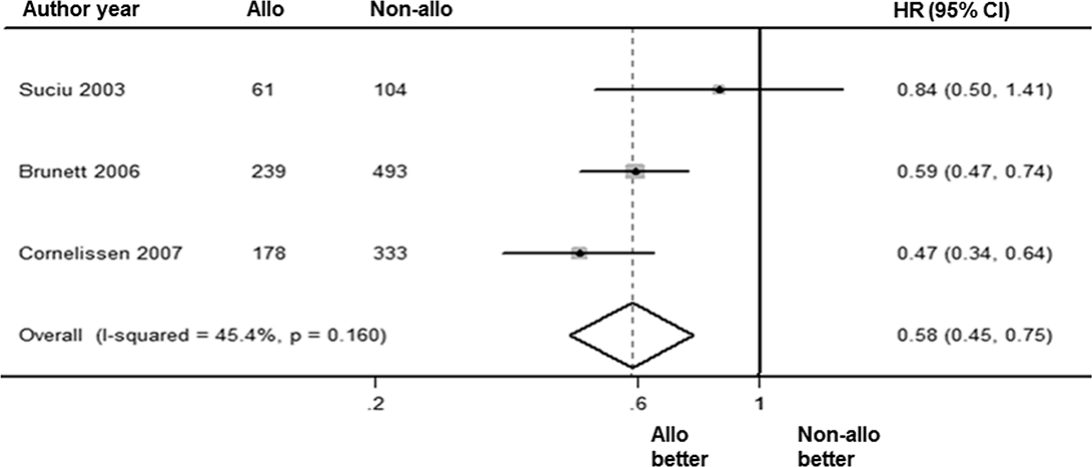
Forest plot of RR benefit of alloHSCT in intermediate-risk AML-CR1.

The RR was analyzed via a random-effects forest plot of the HR from all of the studies. A total of 4 articles reported intermediate-risk AML data for RR. The overall HR was 0.58 [95% CI: 0.45, 0.75] (*P* = 0.000). The overall I^2^ was 45.4% (*P* = 0.16; Fig 5). There was a significant difference between the outcomes of alloHSCT and non-alloHSCT, with fewer patients relapsing following alloHSCT treatment.

### Publication Bias

Publication bias was analyzed used Egger’s funnel test. The plots included 6 articles for determination of the RFS benefit, and 8 articles for the OS benefit. There was no significant difference in publication bias for either of these primary outcomes (*P* = 0.919 and *P* = 0.523, respectively).

## Discussion

Cytogenetic risk profiling is important for stratifying AML treatment. Several clinical trials and meta-analysis have verified that there is no OS or RFS benefit with alloHSCT compared to non-alloHSCT in good-risk AML patients in CR1 [9,60]. While alloHSCT is a curative treatment for AML patients in CR1, it is the first choice for poor-risk AML patients in CR1 [9]. Although Koreth et al. [9] reported that alloHSCT had significant RFS and OS benefit for intermediate-risk AML patients in CR1. As there are limits to the number of patients included in trials and the resulting data, large-scale studies and robust data are urgently needed. To clearly determine whether alloHSCT has RFS and OS benefits for intermediate-risk AML patients in CR1 compared to autoHSCT.

According to the NCCN AML 2014 edition 1 (V1.2014: www.nccn.org) [2], patients with intermediate-risk AML who are age < 60 years of age after post-remission treatment should be enrolled in a clinical trial, receive matched-sibling or alternative donor HSCT, or receive HiAra-C 1–3 g/m^2^ over 3 h every 12 h, on days 1, 3, and 5 × 3–4 cycles. Further clinical trials that compare of alloHSCT versus chemotherapy, especially HiAra-C, are urgently needed.

To address this, we undertook a comprehensive literature search to further and analyze update information on alloHSCT treatment for intermediate-risk AML patients in CR1. The main inclusion criteria of this meta-analysis were that the trials be prospective and controlled. We concluded that alloHSCT produces OS or RFS benefits, which was consistent with the findings of previous studies [9,61]. In comparison with non-alloHSCT, alloHSCT reduced relapse in patients with intermediate-risk AML. Interestingly, further subgroup analysis of alloHSCT versus autoHSCT showed an equal OS benefit. AutoHSCT is considered an alternative treatment to alloHSCT, especially when an HLA-matched related adult donor is not available. Furthermore, alloHSCT has OS benefits compared to chemotherapy, in intermediate-risk AML CR1 patients. The earlier criteria group analysis of OS did not show alloHSCT was superior to non-alloHSCT, however, alloHSCT has OS benefits compared to non-alloHSCT in updated criteria group. It may be related to the inclusive studies are over 25 year times, and there have been some changes in patient population and the clinical management. Notably, the conclusion of RFS was not influenced by the time changes. Limit to the conclusions between two studies in updated criteria group are significant different, the heterogeneity is significant in this group (I^2^ = 57.8%). Large-scale clinical trials are needed.

We considered treatment toxicity by quantifying the results of TRM. While alloHSCT patients benefit from fewer relapses, they may suffer from greater treatment-related toxicity. In our study, that included 3 articles, the I^2^ was 75.4 (*P* = 0.017). The I^2^ was above 50% towing to the fewer studies available to analyze. The high TRM in the alloHSCT group is largely attributable to early mortality. Despite advances in supportive care, and the procedure of alloHSCT improved, the high rate of early mortality is still an important limitation for alloHSCT[62].

Apart from the major variables mentioned above regarding allo-HSCT, There is a consensus on the variables relevant to the success of the procedure, such as the type of transplant performed (myeloablative versus reduced conditioning). Table 2 shows myeloablative conditioning as the only type of conditioning regimens; this may be because patients were adults and the earlier period of clinical trials carried out. Wahid et al. [63] published a meta-analysis showing that there is no OS benefit with myeloablative conditioning regimens over reduced-intensity regimens. Based on the data presented, both myeloablative and reduced conditioning regimens have the same efficacy in intermediate-risk AML adult patients in CR1.

The limitations of this meta-analysis are as follows. First, the majority of the clinical trials included AML patients in CR1, and intermediate-risk AML patients in CR1 comprise but one subgroup this population. Therefore, the original articles only described and compared the characteristics of two groups of AML patients and did not report the specific characteristics of intermediate-risk AML patients in CR1. Second, the various definitions of the intermediate-risk category, including the differing criteria set by SWOG, ISCN, and MRC, included the FLT3-ITD mutation. However, according to the NCCN-AML 2014 version 1 [2], this mutation has been classified under poor-risk.

A meta-analysis is not a discovery tool, but it can help pool evidence and may assist indecision-making when there are no large-scale prospective controlled studies available. Our findings have identified the most appropriate post-remission treatment for intermediate-risk AML based on high quality evidence, and useful for determining the course of future trials. As for TRM, non-alloHSCT provides the greater benefit. These data may help guide decision-making and planning of future trials that compare alloHSCT to either autoHSCT or chemotherapy. It would also be informative to study alloHSCT using a less intensive conditioning regimen in the present era.

## Supporting Information

S1 PRISMA Checklist PRISMA preferred reporting items for meta-analyses checklist. (DOC)

S1 Table Summary of study relating alloHSCT benefit for AML in CR1. (DOCX)
