# Supplementary material for: Efficacy of Allogeneic Hematopoietic Stem Cell Transplantation in Intermediate-Risk Acute Myeloid Leukemia Adult Patients in First Complete Remission: A Meta-Analysis of Prospective Studies
Source: PLoS One. 2015 Jul 21;10(7):e0132620. doi: 10.1371/journal.pone.0132620 (PMC4510363; doi:10.1371/journal.pone.0132620)
Supplement: S1 Table — (DOCX) [file pone.0132620.s002.docx]

Supplemental data for *PLoS One*

**S1 Table. Summary of study relating alloHSCT benefit for AML in CR1.**

| **Author Publication y** | **Trial Name** | **N** | **Enrollment ys** | **Inclusion**  **diseases** | **Comparable arm** | **AlloHSCT benefit in AML-CR1?**  **Study Conclusions** |
| --- | --- | --- | --- | --- | --- | --- |
| Champlin 1985[[1](#_ENREF_1)] | NA | 67 | 1974-1984 | AML | Allo v CC | Overall: RFS-NR; OS-No  Cyto stratified: NA |
| Conde 1988[[2](#_ENREF_2)] | NA | 39 | 1982-1996 | AML CR1 | Allo v CC | Overall: RFS-No; OS-NR  Cyto stratified: NA |
| Reiffers 1989[[3](#_ENREF_3)] | NA | 52 | 1984-1986 | AML | Allo v Auto v CC | Overall: RFS-Yes; OS-NA  Cyto stratified: NA |
| Lowenberg 1990[[4](#_ENREF_4)] | HOVON | 53 | 1984-1987 | AML | Allo v Auto | Overall: RFS-No; OS-No  Cyto stratified: NA |
| Ferrant 1991[[5](#_ENREF_5)] | NA | 96 | 1985-1990 | AML | Allo v Auto | Overall: RFS-Yes; OS-NA  Cyto stratified: NA |
| Cassileth 1992[[6](#_ENREF_6)] | E3483 | 83 | NA | AML | Allo v CC | Overall: RFS-No; OS-NA  Cyto stratified: NA |
| Schiller 1992[[7](#_ENREF_7)] | ALP3-4 | 82 | 1982-1990 | AML | Allo v CC | Overall: RFS-No; OS-No  Cyto stratified: NA |
| Archimbaud 1994[[8](#_ENREF_8)] | LYLAM85 | 58 | 1985-1990 | AML | Allo v CC | Overall: RFS-No; OS-No  Cyto stratified: NA |
| Labar 1994[[9](#_ENREF_9)] | NA | 122 | 1984-NA | AML | Allo v CC | Overall: RFS-Yes; OS-NA  Cyto stratified: NA |
| Hewlett 1995[[10](#_ENREF_10)] | S8125 | 163 | 1982-1986 | AML | Allo v CC | Overall: RFS-No; OS-No  Cyto stratified: NA |
| Zittoun 1995[[11](#_ENREF_11)] | EORTC | 343 | 1986-1993 | AML | Allo or Auto v CC | Overall: RFS-Yes; OS-No  Cyto stratified: NA |
| Sierra 1996[[12](#_ENREF_12)] | CETLAM88 | 115 | 1988-1993 | AML | Allo v Auto | Overall: RFS-No; OS-No  Cyto stratified: NA |
| Keating 1998[[13](#_ENREF_13)] | EORTC/GIMEMA-AML8A | 672 | 1986-1993 | AML | Allo v Auto v CC | Overall: RFS-Yes; OS-No  Cyto stratified: NA |
| Cassileth 1998[[14](#_ENREF_14)] | NA | 740 | 1990-1995 | AML | Allo v Auto v CC | Overall: RFS-No; OS-Yes;  Cyto stratified: NA |
| Brunet 2004[[15](#_ENREF_15)] | CETLAM94 | 144 | 1994-1999 | AML | Allo v Auto (Non favorable risk) | Overall: NA (alloHSCT option for non-fav risk) Cyto stratified: RFS-No; OS-No |
| Jourdan 2005[[16](#_ENREF_16)] | BGMT 84/87/91/95 | 472 | 1984-2001 | AML | Allo v Auto ± v CC | Overall: RFS-Yes; OS-No  Cyto stratified: RFS-NA; OS-NA |
| Schlenk 2008[[17](#_ENREF_17)] | AMLHD93/95/98A/99 | 208 | 1993-2004 | AML | Allo v Auto/CC | Overall: NA (alloHSCT option for non-fav risk) Cyto stratified: RFS-No (favorable-risk); OS-NA |
| Mohty 2009[[18](#_ENREF_18)] | NA | 95 | 1999-2003 | AML | Allo v Auto v CC | Overall: OS-Yes; RFS-Yes  Cyto stratified: NA |
| Basara 2009[[19](#_ENREF_19)] | OSHO AML96/02 | 138 | 1996-2006 | Poor-risk AML | Allo v Auto/CC | Overall: NA (alloHSCT option for poor-risk) Cyto stratified: RFS-Yes; OS-Yes (poor-risk) |
| Hospital 2010[[20](#_ENREF_20)] | ALFA | 205 | 1990-1996 1999-2006 | Poor-risk AML | Allo v non-alloHSCT | Overall: NA (alloHSCT option for poor-risk) OS-No, RFS-No |
| Schlenk 2010[[21](#_ENREF_21)] | AMLHD98A | 267 | 1998-2004 | Poor-risk AML | Allo v CC or Auto | Overall: NA (alloHSCT option for high risk) Cyto stratified: OS-Yes |
| Sakamaki 2010[[22](#_ENREF_22)] | JALSG AML97 | 165 | 1997-2001 | Int/poor JALSG risk | Allo v CC | Overall: NA (alloHSCT for non-fav risk) Cyto stratified: RFS-Yes; OS-No |
| Stelljes 2011[[23](#_ENREF_23)] | AMLCG 99 | 90 | 1999-2007 | Poor-risk AML | Allo v Auto | Overall: NA  Cyto stratified: RFS-Yes; OS-Yes (poor-risk) |
| Cornelissen 2011[[24](#_ENREF_24)] | HOVON/SAKK AML29/42/42A/92 | 1105 | 1987-NA | AML | Allo v Auto v CC | Overall: RFS-Yes; OS-Yes  Cyto stratified: NA |
| Juliusson 2011[[25](#_ENREF_25)] | Swedish Adult Acute Leukemia Registry | 516 | 1997-2006 | AML | Allo v non-alloHSCT | Overall: RFS-NA; OS-Yes  Cyto stratified: NA |
| Huang 2012[[26](#_ENREF_26)] | NA | 132 | 2006-2010 | Int, poor-AML | Allo v CC | Overall: RFS-Yes; OS-Yes |
| Hospital 2012[[27](#_ENREF_27)] | ALFA-9000 | 107 | 1990-1996 | Poor-risk AML | Allo v non-alloHSCT | Overall: NA (alloHSCT for poor-risk) RFS-Yes; OS-Yes |
| Kayser 2012[[28](#_ENREF_28)] | AMLSG | 82 | 1993-2008 | MK adult AML | MRD v MUD v CC | RFS-NA; OS-No |
| Gorin 2013[[29](#_ENREF_29)] | NA | 375 | 2007-2011 | AML | Haplo-allo v Auto | Overall: RFS-No; OS-Yes  Cyto stratified: NA |
| Mohr 2013[[30](#_ENREF_30)] | SAL | 143 | 1996-2009 | abnl(17p) AML | Allo v CC | Overall: RFS-NA, OS-No  Cyto stratified: NA |
| Yoon 2013[[31](#_ENREF_31)] | NA | 163 | 2000-2009 | Poor-risk and  undefined karyotype AML | Allo v Auto | Overall: NA  Cyto stratified: RFS-NA; OS-No |
| Schlenk 2013[[32](#_ENREF_32)] | HOVON04(A),  HOVON29/SAKK30/95,  HOVON42(A)/SAKK30/00,  HOVON92/SAKK30/08;  AML HD93, AML HD98A, AMLSG 07-04 | 124 | 1987–2009 | double mutant CEBPA | Allo v CC v Auto | Overall: NA Cyto stratified: RFS-Yes (favorable-risk); OS-No |

NA indicates not applicable; Allo, allogeneic stem cell transplantation; CC, consolidation chemotherapy; Auto, autologous stem cell transplantation; Int, intermediate; MK, monnosomal karyotype; MRD, matched related donor; MUD, matched unrelated donor; and Haplo-allo, haploidentical allogeneic.

**References**

1. Champlin RE, Ho WG, Gale RP, Winston D, Selch M, Mitsuyasu R, et al. (1985) Treatment of acute myelogenous leukemia. A prospective controlled trial of bone marrow transplantation versus consolidation chemotherapy. Ann Intern Med 102: 285-91.

2. Conde E, Iriondo A, Rayon C, Richard C, Fanjul E, Garijo J, et al. (1988) Allogeneic bone marrow transplantation versus intensification chemotherapy for acute myelogenous leukaemia in first remission: a prospective controlled trial. Br J Haematol 68: 219-26.

3. Reiffers J, Gaspard MH, Maraninchi D, Michallet M, Marit G, Stoppa AM, et al. (1989) Comparison of allogeneic or autologous bone marrow transplantation and chemotherapy in patients with acute myeloid leukaemia in first remission: A prospective controlled trial. British Journal of Haematology 72: 57-63.

4. Lowenberg B, Verdonck LJ, Dekker AW, Willemze R, Zwaan FE, De Planque M, et al. (1990) Autologous bone marrow transplantation in acute myeloid leukemia in first remission: Results of a Dutch prospective study. Journal of Clinical Oncology 8: 287-94.

5. Ferrant A, Doyen C, Delannoy A, Cornu G, Martiat P, Latinne D, et al. (1991) Allogeneic or autologous bone marrow transplantation for acute non-lymphocytic leukemia in first remission. Bone Marrow Transplant 7: 303-9.

6. Cassileth PA, Andersen JW, Bennett JM, Harrington DP, Hines JD, Lazarus HM, et al. (1992) Escalating the intensity of post-remission therapy improves the outcome in acute myeloid leukemia: the ECOG experience. The Eastern Cooperative Oncology Group. Leukemia 6 Suppl 2: 116-9.

7. Schiller GJ, Nimer SD, Territo MC, Ho WG, Champlin RE, Gajewski JL. (1992) Bone marrow transplantation versus high-dose cytarabine-based consolidation chemotherapy for acute myelogenous leukemia in first remission. Journal of Clinical Oncology 10: 41-6.

8. Archimbaud E, Thomas X, Michallet M, Jaubert J, Troncy J, Guyotat D, et al. (1994) Prospective genetically randomized comparison between intensive postinduction chemotherapy and bone marrow transplantation in adults with newly diagnosed acute myeloid leukemia. Journal of Clinical Oncology 12: 262-7.

9. Labar B, Mrsic M, Nemet D, Bogdanic V, Radman I, Boban D, et al. (1994) Allogenic bone marrow transplantation versus chemotherapy for patients with acute myelogenous leukaemia in first remission. Transplantationsmedizin: Organ der Deutschen Transplantationsgesellschaft 6: 235-9.

10. Hewlett J, Kopecky KJ, Head D, Eyre HJ, Elias L, Kingsbury L, et al. (1995) A prospective evaluation of the roles of allogeneic marrow transplantation and low-dose monthly maintenance chemotherapy in the treatment of adult acute myelogenous leukemia (AML): A Southwest oncology group study. Leukemia 9: 562-9.

11. Zittoun RA, Mandelli F, Willemze R, de Witte T, Labar B, Resegotti L, et al. (1995) Autologous or allogeneic bone marrow transplantation compared with intensive chemotherapy in acute myelogenous leukemia. European Organization for Research and Treatment of Cancer (EORTC) and the Gruppo Italiano Malattie Ematologiche Maligne dell'Adulto (GIMEMA) Leukemia Cooperative Groups. N Engl J Med 332: 217-23.

12. Sierra J, Brunet S, Granena A, Olive T, Bueno J, Ribera JM, et al. (1996) Feasibility and results of bone marrow transplantation after remission induction and intensification chemotherapy in de novo acute myeloid leukemia. Catalan Group for Bone Marrow Transplantation. J Clin Oncol 14: 1353-63.

13. Keating S, De Witte T, Suciu S, Willemze R, Hayat M, Labar B, et al. (1998) The influence of HLA-matched sibling donor availability on treatment outcome for patients with AML: An analysis of the AML 8A study of the EORTC Leukaemia Cooperative Group and GIMEMA. British Journal of Haematology 102: 1344-53.

14. Cassileth PA, Harrington DP, Appelbaum FR, Lazarus HM, Rowe JM, Paietta E, et al. (1998) Chemotherapy compared with autologous or allogeneic bone marrow transplantation in the management of acute myeloid leukemia in first remission. New England Journal of Medicine 339: 1649-56.

15. Brunet S, Esteve J, Berlanga J, Ribera JM, Bueno J, Marti JM, et al. (2004) Treatment of primary acute myeloid leukemia: Results of a prospective multicenter trial including high-dose cytarabine or stem cell transplantation as post-remission strategy. Haematologica 89: 940-9.

16. Jourdan E, Boiron JM, Dastugue N, Vey N, Marit G, Rigal-Huguet F, et al. (2005) Early allogeneic stem-cell transplantation for young adults with acute myeloblastic leukemia in first complete remission: An intent-to-treat long-term analysis of the BGMT experience. Journal of Clinical Oncology 23: 7676-84.

17. Schlenk RF, Dohner K, Krauter J, Frohling S, Corbacioglu A, Bullinger L, et al. (2008) Mutations and treatment outcome in cytogenetically normal acute myeloid leukemia. N Engl J Med 358: 1909-18.

18. Mohty M, de Lavallade H, El-Cheikh J, Ladaique P, Faucher C, Furst S, et al. (2009) Reduced intensity conditioning allogeneic stem cell transplantation for patients with acute myeloid leukemia: Long term results of a 'donor' versus 'no donor' comparison. Leukemia 23: 194-6.

19. Basara N, Schulze A, Wedding U, Mohren M, Gerhardt A, Junghanss C, et al. Early related or unrelated haematopoietic cell transplantation results in higher overall survival and leukaemia-free survival compared with conventional chemotherapy in high-risk acute myeloid leukaemia patients in first complete remission Leukemia, 2009:635-40.

20. Hospital MA, Thomas X, Castaigne S, Raffoux E, Maury S, Gardin C, et al. (2010) Long-term outcome associated with current allogeneic stem cell transplantation procedures in younger adults with adverse-risk AML in first CR - A real-life transplant versus no-transplant analysis of the acute leukemia french association (ALFA). Blood 116.

21. Schlenk RF, Dohner K, Mack S, Stoppel M, Kiraly F, Gotze K, et al. (2010) Prospective evaluation of allogeneic hematopoietic stem-cell transplantation from matched related and matched unrelated donors in younger adults with high-risk acute myeloid leukemia: German-Austrian trial AMLHD98A. Journal of Clinical Oncology 28: 4642-8.

22. Sakamaki H, Miyawaki S, Ohtake S, Emi N, Yagasaki F, Mitani K, et al. (2010) Allogeneic stem cell transplantation versus chemotherapy as post-remission therapy for intermediate or poor risk adult acute myeloid leukemia: Results of the JALSG AML97 study. International Journal of Hematology 91: 284-92.

23. Stelljes M, Beelen DW, Braess J, Sauerland MC, Heinecke A, Berning B, et al. (2011) Allogeneic transplantation as post-remission therapy for cytogenetically high-risk acute myeloid leukemia: Landmark analysis from a single prospective multicenter trial. Haematologica 96: 972-9.

24. Cornelissen JJ, Gratwohl A, Van Montfort KGM, Pabst T, Maertens J, Beverloo HB, et al. (2011) Allogeneic Hematopoietic Stem Cell Transplantation (alloHSCT) improves outcome as compared to conventional consolidation in patients aged 40-60 years with AML in CR1 with apparent greater benefit for reduced intensity rather than myeloablative conditioning. Blood 118.

25. Juliusson G, Karlsson K, Lazarevic VL, Wahlin A, Brune M, Antunovic P, et al. (2011) Hematopoietic stem cell transplantation rates and long-term survival in acute myeloid and lymphoblastic leukemia: Real-World Population-Based Data from the Swedish Acute Leukemia Registry 1997-2006. Cancer 117: 4238-46.

26. Huang XJ, Zhu HH, Chang YJ, Xu LP, Liu DH, Zhang XH, et al. (2012) The superiority of haploidentical related stem cell transplantation over chemotherapy alone as postremission treatment for patients with intermediate- or high-risk acute myeloid leukemia in first complete remission. Blood 119: 5584-90.

27. Hospital MA, Thomas X, Castaigne S, Raffoux E, Pautas C, Gardin C, et al. (2012) Evaluation of allogeneic hematopoietic SCT in younger adults with adverse karyotype AML. Bone Marrow Transplantation 47: 1436-41.

28. Kayser S, Zucknick M, Dohner K, Krauter J, Kohne CH, Horst HA, et al. (2012) Monosomal karyotype in adult acute myeloid leukemia: Prognostic impact and outcome after different treatment strategies. Blood 119: 551-8.

29. Gorin NC, Labopin M, Ciceri F, Piemontese S, Arcese W, Di Bartolomeo P, et al. (2013) T repleted haploidentical mismatch allogeneic versus autologous hematopoietic stem cell transplantation in adult patients with acute leukemia in complete remission (CR): A pair-matched analysis from the acute leukemia working party of EBMT. Blood 122.

30. Mohr B, Schetelig J, Schäfer-Eckart K, Schmitz N, Hänel M, Rösler W, et al. Impact of allogeneic haematopoietic stem cell transplantation in patients with abnl(17p) acute myeloid leukaemia British journal of haematology, 2013:237-44.

31. Yoon JH, Kim HJ, Shin SH, Yahng SA, Cho BS, Eom KS, et al. (2013) Normal karyotype mosaicism in adult AML patients with adverse-risk and undefined karyotype: Preliminary report of treatment outcomes after hematopoietic stem cell transplantation. International Journal of Hematology 97: 773-81.

32. Schlenk RF, Taskesen E, Van Norden Y, Krauter J, Ganser A, Bullinger L, et al. (2013) The value of allogeneic and autologous hematopoietic stem cell transplantation in prognostically favorable acute myeloid leukemia with double mutant CEBPA. Blood 122: 1576-82.
